# Asymptotic burnout and homeostatic awakening: a possible solution to the Fermi paradox?

**DOI:** 10.1098/rsif.2022.0029

**Published:** 2022-05-04

**Authors:** Michael L. Wong, Stuart Bartlett

**Affiliations:** ^1^ Earth and Planets Laboratory, Carnegie Institution for Science, Washington, DC 20015, USA; ^2^ Division of Geological and Planetary Sciences, California Institute of Technology, Pasadena, CA 91125, USA

**Keywords:** Fermi paradox, homeostasis, major transitions, dataome, sustainability, extraterrestrial intelligence

## Abstract

Previous studies show that city metrics having to do with growth, productivity and overall energy consumption scale *superlinearly*, attributing this to the *social* nature of cities. Superlinear scaling results in crises called ‘singularities’, where population and energy demand tend to infinity in a finite amount of time, which must be avoided by ever more frequent ‘resets’ or innovations that postpone the system's collapse. Here, we place the emergence of cities and planetary civilizations in the context of major evolutionary transitions. With this perspective, we hypothesize that once a planetary civilization transitions into a state that can be described as one virtually connected global city, it will face an ‘asymptotic burnout’, an ultimate crisis where the singularity-interval time scale becomes smaller than the time scale of innovation. If a civilization develops the capability to understand its own trajectory, it will have a window of time to affect a fundamental change to prioritize long-term homeostasis and well-being over unyielding growth—a consciously induced trajectory change or ‘homeostatic awakening’. We propose a new resolution to the Fermi paradox: civilizations either collapse from burnout or redirect themselves to prioritizing homeostasis, a state where cosmic expansion is no longer a goal, making them difficult to detect remotely.

## 1. Introduction

### 1.1. Life as a feedback between flows of information and energy

Life can be characterized as systems where fluxes of mass and energy lead to the production, transmission and utilization of functional information. This information is said to be ‘functional’ in that it allows the living system to survive over time through a combination of homeostatic mechanisms and autocatalytic behaviour (e.g. [1–3]). Hence, there is a tightly woven dynamic interplay between free energy and information in life: free energy allows information to be encoded, stored and used, and information channels external and internal flows of energy to promote the system's persistence.

Similarly, evolution has been characterized by a series of innovations in the units undergoing selection, energy transduction and information processing. Szathmáry & Maynard-Smith [4] argued that the complexification of life over deep time can be attributed to a series of ‘major transitions’ in the characteristic units of selection acting within biological systems. Such transitions thus represent major shifts in the ways in which biological information is encoded and exploited. So too has human society been shaped and reshaped by innovations that accelerate and widen the spread of information—most notably the inventions of the printing press, telecommunication, computers and the internet. Throughout its evolutionary history, life has learned to exploit different free energy sources in its environment, from geochemical energy to sunlight to the potential energy stored by previous life in flesh and fossil fuels [5]. Within the past 100 years, human civilization has also unlocked nuclear energy and learned to transduce various ‘green’ energy sources (e.g. geothermal, wave motion and wind) into electric power.

Advances in information processing and energy transduction reinforce one another autocatalytically: a greater ability to learn engenders exploration and innovation that opens new free energy resources; greater free energy resources allow for the transition to new information-processing paradigms. These evolutionary leaps sometimes involve the creation of new levels of biological organization and hence new units of selection, e.g. endosymbiosis, multi-cellularity and the formation of social colonies.

### 1.2. Where do we fit in? The rise of human civilization and the dataome

When optimization processes form tight positive feedback loops, the rate of innovation and discovery (and the concomitant demand on energy resources) can quicken dramatically. We see this in the trajectory of human society since the emergence of the ‘dataome’. The dataome encompasses the external recording and processing of information (in e.g. books, architecture and computers) as well as the coevolution of those infological organisms atop of a collection of biological organisms, and it may represent the most recent major transition in information processing—one that we are not only witnessing but also partaking in today [6].

The acceleration of the dataome was intimately tied to the Agricultural Revolution: an energy transition from hunter–gatherer societies to farming, enabled by innovations such as animal husbandry and pottery (e.g. [7,8]). Together, the energy excess afforded by agriculture and the growth of the dataome set the stage for the creation of cities—defined generally as permanent, dense settlements where individuals share resources, infrastructure and ideas—laying the foundation for human civilization as we know it.

In some ways, a city is a superagent composed of individual human agents analogous to a multi-cellular organism that is a superagent composed of individual cellular agents. Both collectives support the differentiation of individual agents (often phrased as a ‘division of labour’), and in both cases, individual agents may come and go while the whole superagent retains its identity. However, there are some key differences between these two organizational structures of life that have been quantified in part thanks to advances in network and complexity science (e.g. [9]).

### 1.3. Cities as a new universality class exhibiting unbounded growth

Recently, cities have been studied as socio-biological phenomena that can be described quantitatively to reveal universal trends that reflect the underlying principles of their organization. Bettencourt *et al*. [10] showed that many properties of cities—GDP, patents, wages, disease and crime—are power law functions of population size with a *super*linear scaling exponent *β* > 1, rendering increasing returns with increasing size. This places cities in a distinct universality class from biological metabolism, which scales as a function of size with a *sub*linear scaling exponent *β* < 1. Other aspects of cities—like number of gasoline stations, length of electrical cables and total road surface—do scale sublinearly; these are associated with material and energy flows and are therefore analogous to metabolism in biology.

Sublinear scaling leads to economies of scale—enhanced efficiency of matter and energy processing, a necessary feature for selective favourability of increased organism size—and a *decreasing* pace of life with increasing size. Examples from biology include a slower metabolic rate in more massive animals, like elephants, than smaller ones, like mice [11].

On the other hand, superlinear scaling results in an *increasing* pace of life with increasing size. A striking example is that the average walking speed is higher in larger cities [12]. So too with crime rates [13] and the spread of infectious diseases [14]. Importantly, the total energy consumption of cities scales superlinearly [10].

The superlinear scaling of seemingly unrelated city properties is attributed to the *social* nature of cities. Indeed, the observed values of this exponent for social characteristics (*β* ∼ 1.2) can be estimated by how the number of effective contacts per capita increases with city size ([10], electronic supplementary material). In other words, in cities, social interactions between agents create a dynamical network of information flow that is hypothesized to drive quantifiable changes in human behaviour that underlie the observed scaling laws [10].

Because growth and wealth production scale superlinearly, cities are highly resilient dynamical entities. Throughout modern history, few cities have completely perished, despite two having had devastating atomic weapons used against them, and despite the coming and going of higher level power structures (e.g. empires, nation states and political parties) [15].

Bettencourt *et al*. [10] developed a growth equation for cities, reproduced below:

1.1 $$\frac{dN(t)}{dt}=\left(\frac{Y_{0}}{E}\right)N{\left(t\right)}^{\beta }-\left(\frac{R}{E}\right)N(t),$$

where d*N/*d*t* is the population growth rate, *Y* represents resources required by the population, *R* is the average resources it requires to maintain an individual and *E* is the average resources it requires to add a new individual to the population. Bettencourt *et al.* [10] generalized the equation further by allowing *R*, the maintenance energy per capita, to vary (see their electronic supplementary material for details), but the results are qualitatively the same.

**Figure 1.**
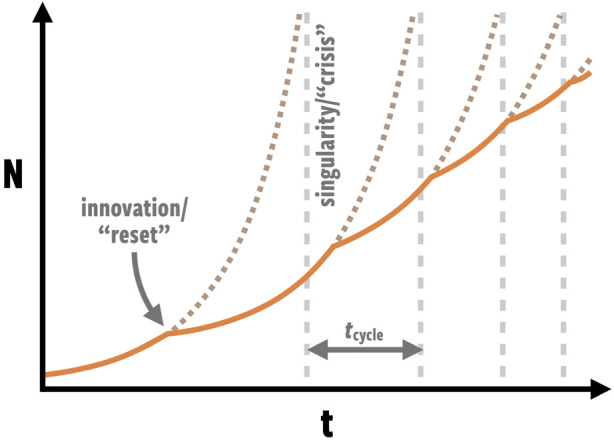
The growth trajectory of a city with scaling coefficient *β* > 1 leads to singularities (dashed vertical lines) that must be avoided by innovative resets. The time scale between resets decreases as the population, *N*, increases. Adapted from Bettencourt *et al*. [10].

Their analysis showed that when *β* > 1, unbounded growth will occur, leading to infinite population (and hence infinite demand on resources) in a finite time. If such a ‘singularity’ is approached unchecked, the system will eventually exceed its energy supply and collapse (or significantly regress). However, Bettencourt *et al*. [10] suggested that singularities can be avoided by major qualitative changes, or ‘innovations’, that reset the initial conditions and parameters of the growth equation and set the city on a new trajectory. If the underlying dynamics remain, meaning *β* > 1 still holds true, then the city will be on a trajectory towards a new singularity later in time (figure 1).

The general growth equation of Bettencourt *et al*. [10] predicts that the time interval between resets, *t*_cycle_, decreases as the population grows:

1.2 $$t_{\mathrm{cycle}}\approx \frac{1}{N_{i}{\left(0\right)}^{\beta -1}},$$

where *N_i_*(0) is the new initial condition after a new innovation occurs. In other words, the frequency of innovations must increase with time. This is consistent with the increasing frequency of population growth cycles and technological change.

## 2. The asymptotic burnout hypothesis

### 2.1. A virtually connected world as a global city

Today, the dataome is omnipresent in our daily lives: portable computers and smartphones bind people across the globe in a digital web of information. The internet—social media in particular—allows individuals who have never met physically to engage in faster, more frequent and more prolonged social interactions. Technology-driven globalization is not only metaphorically making the world smaller, it is quite literally sidestepping the physical limitations of human interactions and allowing people to exchange ideas and influence one another from previously impossible distances at previously impossible rates.

Owing to how deeply intertwined the dataome and human biome has become, it is possible that we are in the midst of another major informational phase transition: one that pushes civilization into a state where the physical colocation of humans in cities is no longer the dominant constraint on human interaction. (Anecdotally, this is consistent with migration away from densely populated city centres to surrounding suburban regions during the COVID-19 pandemic, when work-from-home practices were adopted [16–18].) Liberated by the virtual interactions enabled by information technology, the world may behave as one global city, even if physical cities only cover a small fraction of the planetary surface.

Because it is human interactions that appear to give rise to the superlinear scaling laws of cities, if civilization transitions into a state that can be described as one virtually connected globalized city, it is likely that such an organizational structure will exist in the same universality class as cities. In other words, we conjecture that a technologically connected civilization's productivity, growth and resource consumption would be characterized by scaling laws with a scaling exponent *β* > 1.

As the dataome expands, the preponderance of information processing occurring at the societal level, rather than the individual level, grows. As advances in artificial intelligence are made, it is also possible that human–human interactions may become less important than human–technology, and eventually technology–technology interactions. While human–human interactions are constrained by time and cognitive capacity—the so-called ‘information saturation’ of human experience [19]—the ability for technological agents to interact with one another in an ever-growing digital planetary network could be boundless. What effect these near-future shifts may have on the scaling coefficient of a globalized city remains speculative, but we find it plausible that such transitions could result in an even larger *β*.

### 2.2. Generalizing towards exo-civilizations

The abstract nature of the driving force behind city productivity, growth and total energy consumption—namely, informational exchanges between individual agents—gives promise for the generalization of these principles to hypothetical civilizations elsewhere in the universe. We contend that such principles are likely to be invariant to changes in molecular composition of the lower level agents (e.g. different genetic or metabolic substrates). The emergence and evolution of life on other planets remains speculative at this time. However, it has been argued that certain major transitions—including the onset of life itself—are inevitable, irreversible and ‘reproducible’ (e.g. [20–22]).

Such irreversibility is evident in the ‘core algorithms’ of life on Earth that we observe to stretch back, largely unaltered, in deep time [6]. Examples include the universality of a four-letter genetic code [23,24]; the universality of ATP synthase as a disequilibrium converting engine and ATP as an energy transfer molecule [25,26] and the widespread use of the RuBisCO enzyme in carbon fixation, the gateway for the vast majority of carbon assimilation on present-day Earth [27,28]. Once these innovations emerged, the advantages they confer ‘freeze’ them into the evolutionary threads of life (e.g. [29]), making it difficult for optimization processes such as Darwinian evolution (or hill-climbing on fitness landscapes) to lock in potentially superior alternatives later down the evolutionary line (e.g. [30,31]). While the *material* specifics of life on Earth may be historically contingent, the general principles of life should be universal. Life requires information storage (perhaps not with DNA), free energy transduction (perhaps not with ATP) and a way to assimilate environmental substrates into useful metabolic intermediates (perhaps not with RuBisCO)—but once life discovers how to perform these tasks, such abilities will be strongly selected for and hence persist through time.

Similarly, we can consider higher order features of life—including endosymbiosis, multi-cellularity, language and the creation of a parallel living system in the dataome—to be features locked in by evolution. Although the exact *timing* of such innovations may be stochastic, once they do emerge, they are likely to persist because they become integrated into the positive feedback between information processing and energy transduction described in §1. Hence, we can consider exo-evolutionary trajectories that are similar to our own in that they are governed by the same physical constraints and information–energy feedbacks that have propelled biological evolution on Earth. On these alien worlds, perhaps societies composed of interacting agents will emerge and be subject to the same governing principles as human civilization, even if they do not share our particular material or historical details. At some point in their evolutionary path, societies will likely invent and intertwine themselves with dataomes of their own and may therefore self-organize into a globally connected state characterized by scaling laws with an exponent *β* > 1.

### 2.3. Singularities at the civilization scale

In the scenario laid out above, a global civilization will march towards a singularity where energy resources can no longer sustain the trajectory of unbounded growth. To reiterate, a singularity occurs for trajectories that are headed towards a state of infinite population and energy usage in a finite amount of time. While new energy resources may be discovered, briefly raising the ceiling for total energy consumption, new energy resources cannot sustain a superlinear system indefinitely due to the finite time scale associated with the singularity. Thus, in our view, the important question is not whether new energy resources can be found, but whether innovations (perhaps in energy efficiency or distribution) can reset the system's trajectory by altering the parameters in the growth equation (equation (1.1)). Thus, in this section, we centre our argument around relevant time scales.

As with individual cities, the collapse or regression of planetary civilizations can be momentarily avoided by innovation-related resets that only delay the inevitable. The time scale between resets that must be achieved to maintain viability, *t*_cycle_, decreases over time; in other words, these adaptations must occur at an ever-increasing pace.

Eventually, *t*_cycle_ may become sufficiently short that it exceeds the civilization's time scale for innovation, *t*_innovate_. Perhaps this occurs when *t*_cycle_ ≪ *t*_individual's lifetime_, or the system's learning time scale (driven, in humanity's case, by education). If just one cycle is missed, a regression to a weaker mode of activity will likely occur.

For 2 > *β* > 1 (as is the case for modern cities, where *β* ∼ 1.2 for certain metrics), ∑*t*_cycle_ is a divergent sum, so in theory, a civilization can stay viable forever so long as it meets every single singularity with a reset. However, harmful fluctuations—both external and internal—can threaten the system's stability.

For instance, notable abandoned cities of the ancient world—Angkor, Babylon, Chichen Itza and Pompeii, to name a few—demonstrate that many major early cities succumbed to external fluctuations (e.g. volcanic, climatic or invasion), internal fluctuations (e.g. deforestation or overpopulation) or a combination of the two (e.g. [32–38]). Modern cities have also been abandoned for a variety of reasons: waning economic activity (e.g. California Gold Rush ‘boomtowns’ of the late 1840s and early 1850s), natural disasters (e.g. Craco, Italy, which was evacuated in 1963 after a landslide) and nuclear disaster (e.g. Pripyat, Ukraine, following the Chernnobyl disaster in 1986).

As life grows in free energy-harnessing capability, the magnitude of its internal fluctuations also increases. Examples of energy expansion-related fluctuations include the flooding of the atmosphere with the toxic gas O_2_, due to the energy expansion of oxygenic phototrophy; anthropogenic climate change, driven by the energy expansion of fossil fuels; and the threat of nuclear winter, enabled by the energy expansion of nuclear fission. Informational expansions may also induce catastrophic fluctuations, such as an internet-induced ‘misinformation catastrophe’ in which agents lose their ability to distinguish truth from fiction because innovations in telling truth from lies do not keep pace with the rate at which misinformation becomes more sophisticated. While disease and physical crime may be less malignant in a virtualized global city, viral misinformation and cybercrimes may take their place. Finally, the emergence of any module that produces dissipation and autocatalysis *without* contributing to homeostasis and learning is a dangerous form of ‘sublyfe’ [3] that siphons useful resources away from the whole system; classic examples from biology include cancer and ‘cheaters’ in evolutionary game theory. The recent rise of blockchains and cryptocurrencies, which demand ever-increasing rates of computational resources but do not contribute much to overall stability, may exemplify harmful internal sublyfe fluctuations endemic to dataome–biome symbioses.

Thus, with time, *t*_cycle_ decreases while potentially harmful fluctuations become more likely to derail *t*_innovate_. Once *t*_cycle_ becomes short enough that internal fluctuations or external perturbations can cause *t*_innovate_ > *t*_cycle_ with some non-negligible probability, collapse/regression may be inevitable.

If civilization transitions into a new regime where *β* > 2, perhaps due to a shift where interactions between artificial agents dominate the flow of information, then ∑*t*_cycle_ is a convergent sum (i.e. *t*_cycle_ gets shorter and shorter, such that the cumulative sum converges to an upper bound). In other words, the time scale between innovations vanishes (*t*_cycle_ → 0) in a finite amount of time, and there will be a hard cutoff to the longevity of such a civilization.

In any scenario, it seems that a civilization in which social exchanges between agents form a dynamical network that results in superlinear scaling will eventually reach a point of collapse due to the ever more frequent singularities it faces. We call this concept ‘asymptotic burnout’ and define *t*_burnout_ to be the time scale between the emergence of the dataome and collapse. If collapse were to happen today, and we take the emergence of human language to be the beginning of the dataome (e.g. [39,40]), then humanity's *t*_burnout_ would be on the order of 10^5^ years. If instead we use the emergence of the first cities or the emergence of written language as our starting point (e.g. [41,42]), *t*_burnout_ would be on the order of 10^3^–10^4^ years.

## 3. The homeostatic awakening hypothesis

When burnout occurs, it will necessarily destroy the superagent's *modus operandi* (the ‘way of life’ that drives *β* > 1), but lineages of agents will likely persist, perhaps to self-organize again into cities given enough time. Even if the agents comprising a technological society do not survive long after burnout, it is doubtful that a global civilization's collapse will render its planet completely lifeless, even in the most extreme cases (e.g. nuclear winter). In this scenario, another agent may emerge to establish a new dataome, form the next wave of organized civilization and put itself on a trajectory of unbounded growth, cycles of innovation and eventual burnout.

Can this cycle (indeed, metacycle) of collapse be avoided? At some point prior to *t*_burnout_, the civilization may be able to use its information-processing resources to construct a model of reality that reveals the trajectory that it is on. We define *t*_awakening_ as the time scale between the emergence of the dataome and this realization. We consider the study by Bettencourt *et al*. [10], as well as related works, to be part of our own ‘homeostatic awakening’. Hence, for the case of humanity, Δ*t*_window_ = *t*_burnout_ – *t*_awakening_ is positive. This Δ*t*_window_ is the ‘window’ that a civilization has to purposefully affect some kind of fundamental, systemic change that alters its *modus operandi* away from unbounded growth that results in an arbitrarily large demand on energy in a finite amount of time. Note that there is no *a priori* reason why Δ*t*_window_ must be positive. In many cases, *t*_burnout_ may occur before *t*_awakening_.

As life on Earth has increased in scale and complexity, its ‘cognitive horizon’—defined as the spatial and temporal extent of its predictive abilities and goal-driven behaviour—has increased as well [43]. The cognitive horizon of human civilization is unprecedentedly vast. We can predict the future of physical systems from the subatomic to the cosmological scale, including the behaviour of complex systems, such as humanity itself. It is this predictive power that leads to what we call an awakening—the realization that humanity has been unconsciously optimizing its autocatalytic feedbacks, resulting in the unbounded proliferation and energy exploitation that eventually gave rise to the superlinear scaling laws of cities.

In the one example of life that we have, the cognitive horizon required to understand civilization growth seems to be a product of civilization itself. That is, the mathematical and scientific formalisms that are capable of describing complex systems, as in Bettencourt *et al*. [10], did not emerge at the level of bacteria, biofilms, ant colonies, forests, whale pods or paleolithic hominids; they emerged as a result of the informational transactions allowed when the architecture of human society transitions into cities. This raises the possibility that Δ*t*_window_ is, in general, short compared to, say, evolutionary or geologic time scales.

**Figure 2.**
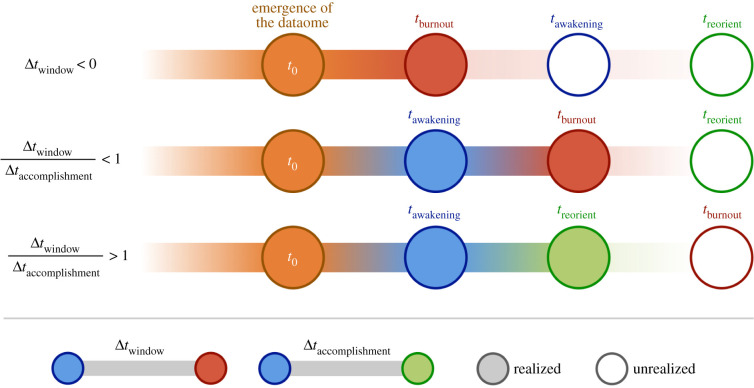
Three scenarios for the evolution of a civilization on a burnout trajectory. *Top*: Δ*t*_window_ < 0. In this scenario, the civilization does not realize its trajectory before it suffers from burnout. *Middle*: Δ*t*_window_ > 0, but Δ*t*_window_/Δ*t*_accomplishment_ < 1. In this scenario, the civilization realizes it is on a trajectory but is unable to accomplish a reorientation towards homeostasis before burnout. *Bottom*: Δ*t*_window_ > 0, and Δ*t*_window_/Δ*t*_accomplishment_ > 1. In this scenario, the civilization is able to both understand that it is on a burnout trajectory and is able to reorient towards prioritizing homeostasis.

In evolutionary histories where Δ*t*_window_ is positive, a civilization has the chance to discover *how* to enact the changes it must make to rearrange its own internal dynamics to consciously prioritize homeostasis. This likely requires a cognitive step beyond simply recognizing the problem of being on a trajectory of unbounded growth. Hence, we define another Δ*t*: Δ*t*_accomplishment_ = *t*_reorient_ – *t*_awakening_, where *t*_reorient_ is the time between the emergence of the dataome and the discovery of how to reorient civilization towards homeostasis. The ratio Δ*t*_window_/Δ*t*_accomplishment_ dictates whether a civilization achieves long-term survival (figure 2).

A redirection would require a rewriting of the fabric of global civilization so that unbounded growth is no longer the priority—or at least no longer the outcome. Because humanity has yet to discover what it must do in order to alter its fate, we are unsure of the cognitive horizon required to reach *t*_reorient_. Many questions must be answered before *t*_reorient_ can be achieved, including: What, besides growth, would we optimize? Does open-ended evolution and novelty discovery require unbounded growth? Can we, as a species, convince ourselves that maximizing a different metric will not only guarantee our persistence, but also improve our overall well-being? Will the dataome play a crucial role in arriving at the solution?

One example of a society actively choosing a non-growth trajectory is the small kingdom of Bhutan, located between India and China. Owing in large part to its pursuit of Buddhist philosophical ideals, Bhutan's government pursues a policy of maximizing ‘Gross National Happiness’ instead of gross domestic product. The achievement of this objective is quantified using an index designed to assess the well-being of Bhutan's population. The ‘four pillars of gross national happiness’ are the following:

(i) sustainable and equitable socio-economic development
(ii) environmental conservation
(iii) preservation and promotion of culture
(iv) good governance.

By placing preservation and conservation front and centre, Bhutan is pioneering a potential path in which material and economic metrics are not necessarily maximized, but other goals such as human and environmental health and the pursuit of knowledge are instead emphasized. Bhutan may of course be an isolated special case that does not in fact demonstrate a society actively choosing an ‘awakened’ path. This country deliberately chose a different objective function to other nations—but whether this has relevance to avoiding burnout is another question. On the one hand, this choice was motivated by sustainability to a large extent, a key part of avoiding exponential growth catastrophes. On the other hand, Bhutan as a country is unlikely to reach any kind of technological singularity in the near-future (burnout risk is relatively low at present).

History contains examples of ‘mini-awakenings’—humans recognizing their own trajectory towards a crisis and consciously learning to adapt, mitigate harmful fluctuations and prioritize homeostasis over unyielding growth. Here, we highlight three:

(i) The mitigation of ozone depletion, caused as an unintentional byproduct of the emission of chlorofluorocarbons, in the second half of the twentieth century. Through legal actions, substances that were found to be harmful to the Earth's ozone layer were banned from use. Today, the trend has reversed and the ozone hole has diminished in size.
(ii) The deescalation of weapons of mass destruction following the Cold War. During the height of the Cold War, the globe's nuclear arsenal exceeded 70 000 warheads, and numerous flashpoints threatened world stability against all-out nuclear warfare. While humanity has not yet removed the threat of nuclear annihilation, today it is somewhat diminished, and the global tally of nuclear warheads stands at less than 14 000 [44].
(iii) The international moratorium on whaling, enacted in 1982. While far from perfect, this moratorium had a fundamental positive impact on the plight of marine mammals. Although whaling was also declining for other reasons, the international recognition of the extinction trajectory caused by whaling in the late twentieth century helped redirect the path to one of conservation and respect. For example, Humpback whale numbers have rebounded significantly since the IWC moratorium and may eventually recover to pre-whaling levels.

Previous evolutionary transitions may also offer a source of optimism. In multi-cellular organisms, neighbouring cells can ‘normalize’ aberrant cells, such as cancerous cells, by altering bioelectric gradients (e.g. [45–47]). Through the regulatory mechanisms of cell-to-cell communication, cells cooperate towards organ-level and organism-level homeostasis, no longer distinguishing between themselves as individual cells but as part of a larger ‘self’ [48–50]. If technological social connectivity is used wisely, perhaps humanity can notice the pain of the world more directly, learn to treat the planet better and wake up to the fact that, on our current trajectory, we're mutually harming ourselves both individually and collectively.

Bettencourt *et al*. [10] showed that when individual organisms self-organize into cities, life transcends into a different universality class than individual organisms. Self-awareness-driven reprioritization towards homeostasis may be the next transcendence that life takes (or must take) after civilization as we know it [51].

## 4. Burnout and awakening in the context of the Fermi paradox

The Fermi paradox asks: in a universe that seems amenable to abiogenesis and the evolution of life leading to technological civilizations, why haven't we seen definitive evidence of extraterrestrial civilizations? We contend that this question presents itself as a ‘paradox’ because there is an implicit assumption that the trajectory of progress can be extrapolated from the past, i.e. that the future is a linear extension of past and current trends. Specifically, civilizations are projected to attain greater energy harnessing capabilities and greater knowledge of the universe until they spread in an unbounded colonialist manner across the galaxy.

The Kardashev scale is one example of this mode of thinking. This measure of the ‘advancedness’ of civilizations is based on the concept of energy expansions: a Type I civilization uses all of the energy available at the planetary scale; a Type II civilization uses all of the energy available at the planetary system scale and a Type III civilization uses all of the energy available at the galactic scale. It has been argued that once a Type II civilization becomes interstellar, it should spread across the galaxy in a relatively short amount of time [52–55]. Hence, the lack of evidence of extraterrestrial visitors is widely considered to be consistent with the belief that a Type III civilization does not currently exist in the Milky Way. Additionally, extragalactic observations suggest that Type III civilizations are rare [56], although this absence may stem from an inability to observe in the appropriate way.

Two important aspects of biology are missing from Kardashev-like frameworks: (i) evolution is not always gradual and linear, but is impacted by major transitions and ‘punctuated equilibria’ and (ii) the dynamics of city-like living systems in which information flows and energy flows result in superlinear scaling and singularity crises.

**Figure 3.**
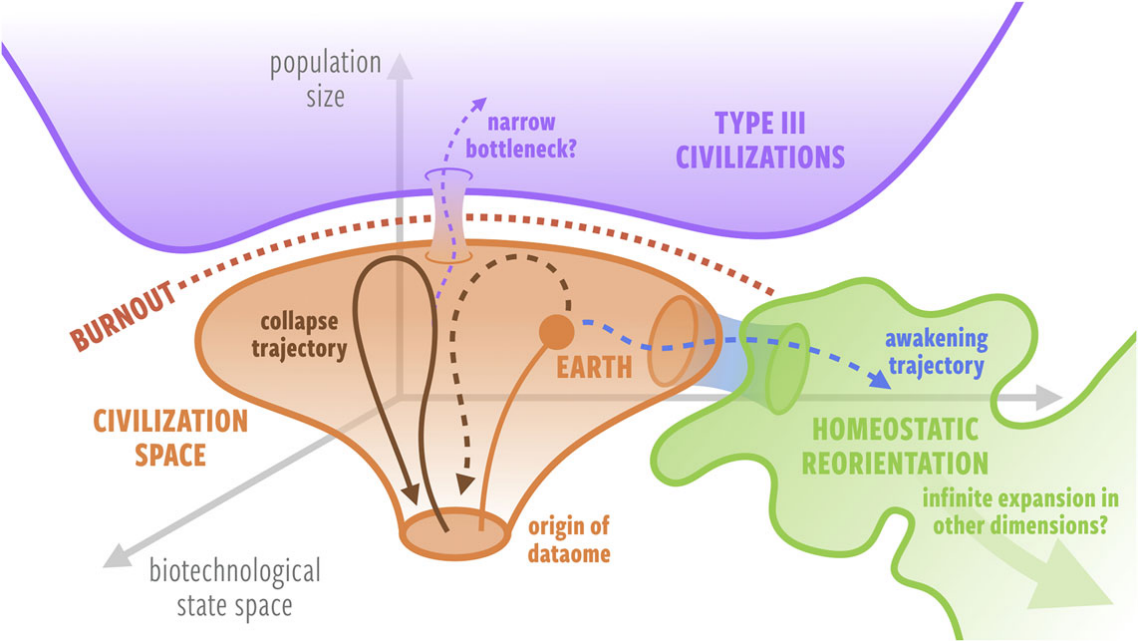
Perhaps hypothetical Type III civilizations are in an inaccessible region of biotechnological–population size state space, where civilization trajectories are bounded by a ‘burnout horizon’, and long-lived civilizations have consciously reoriented their trajectories away from growth in population size and length scales to explore other dimensions of biotechnological state space. Note that we do not rule out the possibility of some kind of channel that might allow a transition to the Type III region.

Perhaps Type III civilizations on the Kardashev scale are in an unreachable part of biotechnological state space because most civilizations face burnout at the planetary/sub-planetary scale. When burnout approaches, if Δ*t*_window_ < 0, the civilization will necessarily collapse or regress until it iterates into a new cycle. If Δ*t*_window_ > 0, the civilization has a chance to consciously transcend into prioritizing homeostasis; otherwise, it will collapse or regress. Hence, there may be very few or no plausible trajectories through biotechnological state space that connect humanity's present state and the region occupied by hypothetical Type III civilizations (figure 3).

Whether homeostatic transcendence happens in all or a minute fraction of all cases is irrelevant to the observables consistent with the Fermi paradox. If collapse or reorientation are the only likely options, then whatever their yields are, a civilization's growth will cease—either because cosmic expansion is no longer viable or because it is no longer an imperative. This argument may provide a theoretical foundation for the ‘sustainabilty solution’ to the Fermi paradox [57].

Other ‘solutions’ to the Fermi paradox involve ‘bottlenecks’ that are probability-based. In the language of the Drake equation [58], this takes the form of equating one of the biotechnical factors to a very low probability. The solution we propose here is of a different kind: it is an *inevitable* barrier, emergent from the dynamics of energy and information flows within a living system, that civilizations will either meet or learn to redirect themselves around. (Note that if the burnout–awakening hypothesis is borne out, this does not preclude the possibility of other ‘great filters’ as well).

**Figure 4.**
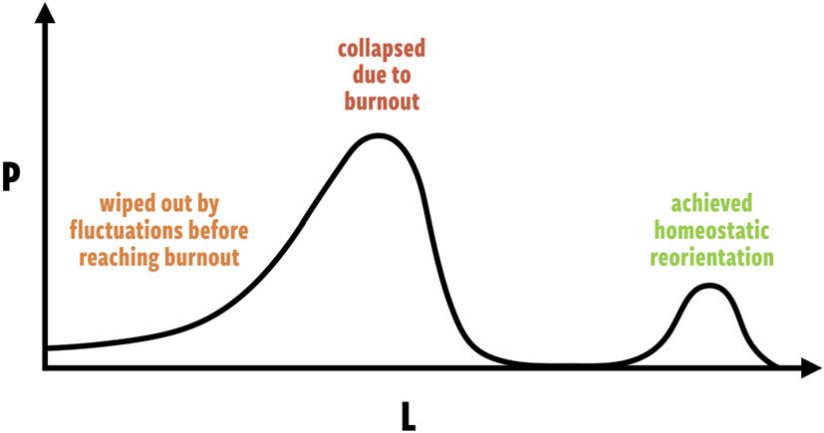
An example bimodal distribution of the lifetime *L* of civilizations. The relative sizes of each mode is dependent on how many civilizations are able to transcend into homeostatic awakening and avoid burnout.

The lifetime of ‘technologically advanced’ civilization, *L*, is considered to be one of, if not the most, uncertain variables in the Drake equation. If the burnout–awakening hypothesis does indeed describe the fate of civilizations across the cosmos, then *L* may have a *bimodal* distribution. The civilizations that encounter burnout will be extremely short lived; the civilizations that consciously choose homeostasis will be significantly longer lived (figure 4).

Jill Tarter (2021 personal communication) has suggested, provocatively, that it might be the detection of other extraterrestrial civilizations that spurs a civilization into the long-lived mode. This suggestion is not incompatible with our idea. Discoveries about the universe, in general, inform our collective psyche. The discovery of alien life, and especially alien intelligence, may play an important role in affecting the monumental changes necessary to complete a planetary civilization's reorientation towards homeostasis.

## 5. Discussion

We have outlined a hypothesis that planetary civilizations, virtually connected by their dataomes, may grow along trajectories toward asymptotic burnout. As burnout approaches, civilizations may attain the cognitive horizon to understand their trajectory and affect a reprioritization towards homeostasis. Either outcome—homeostatic awakening or civilization collapse—would be consistent with the observed absence of Type III civilizations.

The goal of this article is simply to state the burnout–awakening hypothesis and provoke discussion, introspection and future work. Like so many other astrobiological hypotheses, there is no evidence yet that this idea is true, other than its rooting in the laws of life that seem to govern biological organization on Earth.

We acknowledge that the hypothesis laid out above rests on several crucial assumptions, namely:

(i) Bettencourt *et al*. [10]'s universal urban growth equation is a correct description of city growth and also captures the essence of a broader universality class of large-scale biological organizations.
(ii) Bettencourt *et al*. [10]'s argument that the network of social interactions provided by dense urban environments causes some city metrics to have a scaling coefficient *β* > 1 is true.
(iii) When the dataome evolves to allow the network of social interactions to transcend physical location, global civilization can be described by a similar general growth equation with *β* > 1.
(iv) This reasoning can be abstracted to exo-civilizations because it relies on the general idea of social organismal agents self-organizing into city-like superagents. According to the basic ideas of complex systems, the emergence of such high order is expected to be invariant to lower level details.

One major caveat is that perhaps the formation of city-like entities (with *β* > 1) is not a universal feature of life in the universe, but some unique quirk of life on Earth. Astrobiologists often caution against using the specific characteristics of Earthly life to guide our search for life elsewhere, because such details might be single instantiations of, rather than fundamental to, what life is. For example, Kempes & Krakauer [59] argue that replication is just one form of *persistence*, which is the more universal aspect of life. In a biosphere where self-replication of individuals is not the paradigm, evolution would proceed much differently, potentially resulting in different growth equations and macroscopic scaling laws. The idea of cities as an organizational structure of life may not even make sense in such a scenario.

Even if the individual agents in an exo-civilization are human-like in physiology, perhaps it is not appropriate to assume that human-like social structures will necessarily follow. An analogy can be drawn to exo-biochemistries: Because ‘chemical space’ is huge, some astrobiologists have hypothesized that alien biochemistries could take many different forms, and that the particulars of our biochemistry were influenced by the specific chemical environment on the early Earth as well as historical contingencies of our single evolutionary path (e.g. [60–63]). Similarly, perhaps ‘cultural space’ is huge, resulting in technological civilizations whose self-organizing dynamics appear as foreign to us as a lyfe form that doesn't use H_2_O as its solvent.

Assuming that the burnout hypothesis is true, a civilization's path to homeostasis may come in other forms than a cognitive choice. In other words, a conscious awakening, as described in §3, may not be strictly necessary to achieve longevity. Perhaps reorientation towards homeostasis may happen by ‘accident’ or even be a feature of a deterministic trajectory. In one speculative scenario, virtual spaces (social media, video games, etc.) may absorb most, if not all, of the cognitive attention of humanity. When biological agents become physically and mentally immersed in the dataome, civilization might turn ‘inward’ to serve the dataome's priorities, whose persistence could be maintained without unbounded growth.

Life as ‘persistence’ is one view that underwrites the free energy principle (FEP, a.k.a. active inference), namely, that life will exhibit a tendency to minimize surprise under internal model predictions of external environmental states [64]. Under this framework, life restricts the growth of the entropy of its own state(s) by minimizing the cognitive mismatch (surprise) between internal inferential models and the environment. This approach has produced a wide range of intriguing insights, though its theoretical and philosophical foundations continue to be critically assessed [65–67]. It is possible that an FEP-like dynamic underlies the emergence of homeostatic awakening events. The FEP predicts convergence between living systems' cognitive models and the environment, such that the persistence of those living systems is maximized. Hence, the dynamics of homeostasis, allostasis [68,69] and the robust stability of biological systems at different hierarchical levels (e.g. organismal to societal) could be interpreted in terms of the FEP. Note, however, that the divergent feedback dynamics that lead to burnout are beyond the scope of the FEP and require different explanatory frameworks.

How might one imagine testing the asymptotic burnout and homeostatic awakening hypotheses? The most conclusive evidence would take the form of exo-anthropology—the direct observation of extant or extinct exo-civilizations. However, this is not likely to be achieved with present or near-future technologies. Ironically, if the burnout–awakening hypothesis is true, exo-anthropology might be an extraordinarily rare activity across the cosmos, limited to instances where civilizations can identify the signs of burnout or awakening on neighbouring planets that host(ed) life, or the post-burnout remnants of a prior civilization on their planet [70].

The burnout–awakening hypothesis does not preclude the remote detection of exo-civilizations, be they approaching burnout or post-awakening, via planetary-scale technosignatures. Such technosignatures include electromagnetic transmissions (both accidental and purposeful), industrial changes to atmospheric chemistry, the construction of artificial structures in space and the complexity of planetary time series measurements [71–75].

In fact, civilizations that are near burnout may be the *most* detectable exo-civilizations, as they would be altering their environments and dissipating free energy in a wildly unsustainable manner—fluctuations on the planetary scale that exhibit the largest signal-to-noise. This presents the possibility that a good many of humanity's initial detections of extraterrestrial life may be of the *intelligent*, though not yet *wise*, kind. Observing such burnouts (provided humanity is long lived enough to do so) would provide potential confirmation of part of our hypothesis. On the other hand, persistent civilizations that transition through homeostatic awakening may be difficult or impossible to detect. If so, this would be sympatico with Karl Schroeder's recasting of Arthur C. Clarkes’ ‘technology looks like magic’ proposition: ‘Any sufficiently advanced civilization will be indistinguishable from Nature’ [76,77].

Regardless of whether the burnout–awakening hypothesis does or does not describe a *universal* trajectory for life in the universe, it is critical to know whether *humanity* is in danger of suffering from an asymptotic burnout. We hope that future work will test the assumptions outlined above. Specifically, we encourage the collection and analysis of global datasets to quantify how growth, productivity and other social metrics have changed over time. Informational innovations—from printing to radio to the internet—have allowed for faster and more widespread social interactions with less of a need for temporal and spatial synchrony between agents. Can different scaling laws be identified for different stages of dataome evolution? Can modern civilization be treated like one global city driven by virtual social interactions? At what scales do resources become constraining and could yet unrealized energy transitions liberate a civilization from burnout trajectory? We also encourage theoretical work to complement these empirical analyses. A deeper theoretical understanding of the dynamics that determine the value of *β* may reveal suggestions for how we can enact a fundamental change to our ‘way of life’ that helps us consciously avoid self-induced collapse.

## Data Availability

This article has no additional data.
